# Assessment of the incubating environment for investment in biogas technology in Syria by using AHP and SWOT

**DOI:** 10.1007/s10668-023-03137-9

**Published:** 2023-03-27

**Authors:** Ghaith Hasan, Jana Mazancová, Hynek Roubík

**Affiliations:** Department of Sustainable Technologies, Faculty of Tropical AgriSciences, Czech University of Life Sciences Prague, Kamýcka 129, 16500 Prague – Suchdol, Czech Republic

**Keywords:** Biogas technology, Analytic hierarchy process, SWOT analysis, Biogas adoption, Developing countries

## Abstract

In light of the massive energy supply shortage due to the Syrian war since 2011, renewable energy adoption has a high potential to cover the actual energy demand. Hence, this study aims to shed light on the factors that affect investment in biogas technology. With the scarcity of research on alternative energies in Syria, this paper focused on the characteristics of the Syrian environment toward biogas technology adoption. The results show that Syrian society accepts and desires to adopt new technologies, representing an optimal strategy to stimulate biogas technology use and the need to spread awareness about its benefits. The SWOT model was applied to identify strengths, weaknesses, opportunities, and threats facing biogas technology adoption. The analytical hierarchy process model was applied to set priorities and make better decisions related to the knowledge of biogas, acceptance of biogas technology, desire for and common approach for its use, the resulting organic fertilizer, and administrative and financial aspects. The work concludes that the southern region was at the forefront in the areas studied in terms of weights of biogas technology investment criteria, subsequently, the central and later the coastal regions. By presenting a systematic and comprehensive approach, this study represents a roadmap to assist decision-makers in inking decisions related to adopting and deploying biogas technology on a larger scale and contributes to developing a criterion for selecting biogas sites in Syria.

## Introduction

The reality of the Syrian war that has been ongoing since 2011 has cast a shadow over the energy sector. Northeastern Syria contains more than 80% of the country’s energy sources; its exit from the control of the Syrian state, in addition to the sabotage and destruction of electric power plants and gas and oil fields during the war, is among the primary reasons that led to the current energy shortage (Li et al., 2022, Cheung et al., 2020, World Bank, 2022, Li. et al. 2022, SANA, 2022, Hatahet and Shaar, 2021). Furthermore, direct and indirect losses in the oil sector, which amounted to about 100.5 billion USD between 2011 and 2022, caused a severe shortage of various oil derivatives, as 60% of the energy infrastructure was destroyed (SANA, 2022).

Globally, countries are increasingly interested in renewable energy use contributing to greenhouse gas emissions reduction, climate change mitigation, circular economy development, and sustainable energy utilization (D'Adamo et al., 2019, Yazan et al., 2018, Falcone et al., 2018). Syria, in addition, also constitutes its application as a way to solve the problem of acute shortage of energy sources due to the heavy ongoing conflict (Laub, 2020, OCHA, 2017).

When comparing alternative energy projects in Syria with Arab oil countries whose economy is mainly dependent on oil, the share of alternative energy on the total energy supply did not exceed 1% (30 MW) in Syria in 2019 (IRENA, 2021), while UAE production in 2022 amounted to 2.6 GW of alternative energy, especially solar. Saudi Arabia produces 0.78 GW, while Egypt, which hosted the UN Climate Change Conference (COP27) in 2022, even 3.5 GW. Although, in Syria 1.7 GW were produced from alternative energy sources (Behrsin et al., 2022) in 2021; to achieve the 2030 goals to add 2 GW more (1.50 GW wind power, 0.25 GW biomass-based power, 0.25 GW photovoltaic power) (Krepl et al., 2020), it is necessary to highlight the importance of biogas energy in countries that suffer from war effects, such as Syria's case, explore strengths and opportunities and exploit them, and work to overcome obstacles and threats facing the adoption of this technology.

In view of the facts mentioned, it is noticeable that there is a real gap between the declared goals and the results achieved in Syria. There is a dearth of literature related to bioenergy systems adoption in developing countries that are witnessing exceptional circumstances such as civil wars (Yemen, Iraq, Libya, Lebanon, Syria) (Krepl et al., 2020). The SWOT analyzing of biogas technology adoption factors contributes to defining its importance in the achievements of the declared goals.

Previous studies have proven the suitability of various renewable energy resources (solar, wind, biomass, hydropower, tidal, wave and geothermal energy) in the Middle East (Tumenand Caliskan, 2022; Shawon et al., 2013, Alshami and Hussein, 2021; Noorollahi et al., 2019; Salah et al., 2022). The current conditions encouraged local communities to search for alternative solutions to the energy problem. This has been reflected in the spread of home-scale solar energy use for those who can obtain it despite its high price compared to the purchasing power of the individual (Elistratov and Ramadan, 2018, Al Halabi et al., 2021). But surprisingly, biogas production has not received attention despite its high potential in terms of available feedstock, reducing dependence on natural gas and timber and contributing to the high need for sustainable energy in the Syrian countryside (Hasan et al., 2022, Jafar and Awad, 2021).

Historically, the Syrian experience with biogas technology is limited, despite the favorable conditions of sufficient feedstock availability and the moderate climate of the region. Studies (Jafar and Awad, 2021; Abdo et al., 2015) attribute the restricted dissemination primarily to economic, technical and social challenges. Since the 1990s, several small-scale biogas plants have been established by the Ministry of Agriculture and Agrarian Reform, the Arab Center for Studies of Dry Areas and Dry Lands (ACSAD), and the National Center for Energy Research. Therefore, Alafif et al. (2008) and Almikdad et al. (2015) showed that biogas production is a technical solution that is economically and environmentally viable; it allows the use of organic, animal, and plant waste, sewage, and industrial waste and also has additional economic value in the resulting organic fertilizer; it also allows investment of the energy produced in rural communities. A study by Al-Mohamad, A. (2001), showed that the presence of low-cost energy sources that covered the demand in Syria and the high implementation costs of renewable energy projects was among the rationales for the modest application of such projects. Since the onset of the conflict in 2011, international organizations such as FAO and Global Communities have helped to install small-scale biogas plants in poor rural areas (OCHA, 2017, Global Communities, 2018) demonstrating the tendency to adopt alternative energy to fill the energy shortage caused by the war. This is considered the best option due to the availability of ideal conditions for its adoption in the post-war period.

This paper explores the current strengths, weaknesses, opportunities, and threats of the economic environment for biogas technology dissemination in Syria. In addition, it analyzes the common approach and criteria for selecting biogas unit locations. It also defines the best areas to establish biogas units among other regions studied in Syria. Table 1 illustrates the methods previously applied and their intersection with this study.

**Table 1:** Overview of studies that employ the SWOT-AHP approach

Number	Method	Intersection with our study	Country	Reference
1	SWOT AHP	Measuring biogas and biofertilizer production	Nigeria	Audu et al., (2020)
2	SWOT	Considering biogas production as a sustainable development tool	EU	Pawlita et al., (2020)
3	SWOT	Analyzing prospects and challenges of large-scale biogas technology	Bangladesh	Saha et al., (2022)
4	SWOT	Projecting biogas sector development	Latvia	Bumbiere et al., (2021)
5	SWOT	Empowering biogas as renewable energy for sustainable energy evolution	Pakistan	Kamran et al., (2020)
6	AHP	Analyzing the barriers impeding rural domestic biogas plants diffusion	Rwanda	Mukeshimana, et al., (2021)
7	AHP	Assessing biogas production from industrial liquid wastes	Indonesia	Nasution et al., (2020)
8	AHP	Analyzing impact factors of biogas technology implementation in rural areas	India	Yadav et al., (2022)
9	SWOT AHP	Measuring prospects of biogas technology and its contribution to sustainable energy supply	Austria	Brudermann et al., (2015)
10	SWOT AHP	Testing the biomethane and biogas contribution to electricity production	Spain	Fernández-González et al., (2020)
11	MCDA AHP	Locating biogas power plants in energy-poor areas	Thailand	Nantasaksiri et al., (2021)
12	AHP	Determining the factors affecting the generation of biogas from solid waste	Brazil	Ruoso et al., (2022)
13	MCDA AHP	Determining the obstacles and factors affecting the selection of sites for the construction of biogas units	Portugal	Silva et al., (2014)
14	MCDA AHP	Analyzing the best site and size of biogas plants	Turkey	Yalcinkaya et al., (2020)
15	Reflexive thematic analysis	Identifying factors and logic for biogas plants location	Sweden	Feiz et al., (2022)

## Methodology

Primary data collection was carried out through a questionnaire survey among farmers. A standardized paper-based questionnaire was distributed on 300 farms between March 2019 and January 2020. The response rate of 85% (255 farms) covers the Coastal (84 farms), the Central (69 farms), and the Southern (102) regions of Syria. The questionnaire was comprised of five principal chapters covering the following: (i) respondent’s knowledge of biogas (incl. biogas production processes, biogas technology and its costs); (ii) the biogas technology respondent’s real and potential acceptance level; (iii) the respondent’s approach to the use of both biogas and digestate (organic fertilizer); (iv) the attitude of the respondent toward the management of the biogas unit (individual vs. collective, private vs. governmental); and (v) the knowledge and attitude of the respondent about the financial aspects of biogas technology (costs and expected profits).

The collected data were computerized in Microsoft Excel and analyzed in SPSS V20 Statistical Package for Social Sciences Program. Two analytical methods were employed, such as SWOT and AHP.

The methodological approach aimed at reducing potential bias in responses by quota sampling the target groups in seven provinces (Latakia, Tartus, Homs, Hama, Damascus, Sweida and Daraa).

### SWOT analysis

To specify effective strategies for the implementation of biogas technology in Syria, take advantage, empower and work on weak points, and avoid threats, SWOT analysis was used to analyze areas of strength, weakness, opportunity, and threats (Olabi et al., 2022, Longsheng et al., 2022). SWOT analysis is used to obtain a comprehensive view of the study area by analyzing the current and future environment. At the same time, it provides a planning tool for dealing with the changing environment (Kowalska-Pyzalska et al., 2020; Paschalidou et al., 2016; Ng, 2021). In this research, SWOT analysis is used to monitor, evaluate, and disseminate information on the internal and external environment. This leads to an effective strategy that should enhance the strengths and opportunities in the environment studied and reduce the impact of weaknesses and threats.

As a qualitative analysis, SWOT analysis does not deliver precision in terms of the relative importance of relevant factors (Brudermann et al., 2015). Therefore, Analytic Hierarchy Process AHP was employed, which is based on a comparison and weighting of SWOT factors through pairwise comparisons, to find out the most relevant factors within the group (Kurttila et al., 2000).

### Analytic hierarchy process

**Analytic Hierarchy Process (**AHP) is a hierarchical analysis procedural technique widely used for making various types of complex decision in many sectors (Burak et al., 2022; Pathak et al., 2022), introduced by Thomas L Saaty in the 1970s (Saaty, 1977). It has attracted many researchers due to its mathematical properties and the ease of obtaining the data required to use it (Ilbahar et al., 2022). This process is known as the theory of constructing indicator*s* using marital comparisons that adopt the opinion of experts and decision-makers within the limits of a specific scale. It can help the decision-maker to set priorities and make better decisions by transformation the goal into a hierarchical series of criteria arranged in a horizontal and vertical matrix. Within the matrix, each criterion is compared separately in double comparison (Mastrocinque et al., 2020). The method relies on determination of the relative importance of a specific set of criteria and alternatives to a predetermined goal, considering the criteria and sub-criteria. The AHP attempts to introduce analytical thinking into decision-making based on different principals shown below:

1. Composing an order of decision problems.
2. Prioritizing while using Saaty’s numerical scale (Table 2) to weight sub-criteria, criteria, and other alternatives. The weighing procedure was carried out in the Expert Choice Program (Ishizaka and Labib, 2009; Bagheri et al., 2021)
3. Creating a pairwise matrix by summing the outputs of Saaty's scale in one pairwise matrix for each level (Sedghiyan et al., 2021, Gottfried et al., 2018).
  $$X = \left[\begin{array}{ccccccc}1& A1/A2& .& .& .& .& A1/An\\ A2/A1& 1& & & & & \\ A3/A1& & 1& & & & \\ .& & & 1& & & \\ .& & & & 1& & \\ .& & & & & 1& \\ An/A1& An/A2& .& .& .& .& 1\end{array}\right]$$

where Ai (i = 1,2,…,*n*) represents the weight of each factor from the SWOT analysis table.

**Table 2:** Saaty scale summary of the nine-point ratio (based on Mukeshimana et al., 2021; Nilsson et al., 2016)

Name of points	Equal Importance	Weak Importance	Strong importance	Very strong importance	Strongest importance
Description	More than one criterion contributes at the same level to the objective	One criterion is slightly different from the other	One criterion is essentially different from another	One criterion is different from another	One criterion is different over another
Importance intensity	1	3	5	7	9

Average values are used when compromise is needed between the previous values, such as 2 - 4 - 6 - 8

4. Creating the consistency ratio using the normalized eigenvector for each matrix λmax (Yadav et al., 2022).
  $$\mathbf{X}\mathbf{w}={\varvec{\uplambda}}\mathbf{m}\mathbf{a}\mathbf{x}\mathbf{w}$$

X denotes the value of preference vectors. W can be calculated by determining the eigenvector of A and its corresponding to its eigenvalue.

5. Calculating the index of consistency CI:
  $${\varvec{C}}{\varvec{I}}={{\varvec{\uplambda}}\mathbf{m}\mathbf{a}\mathbf{x}-{\varvec{n}}}/{{\varvec{n}}-1}$$
6. Calculating the ratio of consistency CR by comparing the value of the index of consistency CI with that of the index of randomization RI:
  $$\mathbf{CR} =\mathbf{CI}/\mathbf{RI}.$$

where (RI) is the Random Index that relates to the matrix structure Table 3. When the CR is $\le 10\mathrm{\%},$ the matrix consistency is acceptable; otherwise, evaluation should be made again of pairwise comparisons in the matrix (Gottfried et al., 2018). RI is essential in the consistency of the comparison matrix used in the decision-making process (Shyamprasad et al., 2020; Rao et al., 1998; Wedley, 1993). After the above levels, we multiply each element by its corresponding criteria (Saaty, 1977).

**Table 3:** Random index values (Saaty, 1977)

**N**	1	2	3	4	5	6	7	8	9	10
RI	0	0	0.58	0.9	1.12	1.24	1.32	1.41	1.45	1.49

## Results and discussion

### SWOT analysis

Based on the questionnaire, the answers were specified on the 5-point Likert Scale ( (1) Strongly disagree; (2) Disagree; (3) Neither agree nor disagree; (4) Agree; (5) Strongly agree). To identify the SWOT factors and measure the agreement of each statement by calculating the mean score for each SWOT factor code, Table 4 illustrates the strengths of adopting biogas technology in Syria.

**Table 4:** Strengths points formulation for the adoption of biogas technology in Syria

	Factor code	Description	Mean score (5-point Likert scale)*
Strengths	S1	Attention to innovations	4.25
	S2	Biogas technology reduces final waste volume	4.08
	S3	Being prepared for separation of organic waste (kitchen and garden waste) from the rest of the household waste	4.08
	S4	Showing desire to use digestate resulting from biogas technology in the home or farm garden	3.93
	S5	The degradation of organic waste results in a plant fertilizer	3.93
	S6	Being prepared for purchase a biogas unit and use it at home or on the farm	3.75
	S7	Other energy sources are expensive	3.69
	S8	The use of biogas is recommended at the home level	3.65
	S9	The decomposition of organic waste produces fertilizer through biogas technology, liquid and solid waste	3.48
	S10	Support the use of biogas technology at home and with home management only	3.33
	S11	Knowing where to obtain the necessary information in case of interest in biogas technology	3.14
	S12	The ability to store manure	3.11
	S13	The ability to collect dung regularly	3.04
	S14	Receive training in biogas technology	2.19
The total average			3.54

*1—lowest, 5—highest

The total average response of the respondents to the strength dimension was 3.54, which is greater than 3 (which is the neutral scale in the Likert scale analysis). At the level of the paragraphs, paragraph (8) had an average point of 4.25 which is higher than 3, while paragraph (6) was the only Paragraph lower than 3, with an average of 2.19. These results confirm that most of the sampled individuals emphasized the most important strengths enjoyed by the Syrian environment around biogas technology.

Given the content of the factors description, the most important strengths of the Syrian environment in biogas technology are the interest of Syrian farmers in modern technology, their willingness to deal with organic waste, their interest in the results of that process, and their desire to use it on a large scale. The results are consistent with the strengths of the Ugandan environment in terms of the interest of farmers in biogas as a clean and reliable energy that contributes to the effective management of organic waste (Okello et at., 2014). Table 5 shows an assessment of the weakness dimension.

**Table 5:** Weaknesses points formulation for the adoption of biogas technology in Syria

	Factor code	Description	Mean score (5-point Likert scale)*
Weaknesses	W1	The initial construction cost of a biogas unit is high	3.79
	W2	There exist other alternatives better than biogas technology for organic waste treatment	3.07
	W3	Running a biogas plant at home or on the farm will require much time and effort	3.02
	W4	Digestate is the low quality of fertilizer	2.87
	W5	Energy produced from manure is not recommended for cooking	2.67
	W6	Other alternatives to organic waste management are better than biogas technology	2.66
The total average			3.02

*1—lowest, 5—highest

The total average response of the respondents to the weakness dimension reached 3.02, slightly greater than 3. At the same time, for the paragraphs, paragraph (1) had the highest average of 3.79, while paragraph (5) was the lowest paragraph with an average of 2.67. In general, these results confirm that most of the individuals in the sample confirmed the most critical weaknesses that Syrian farmers face in adapting biogas technology.

Given the content of the description of the factors, the most important weaknesses that the Syrian environment suffers from biogas technology are cost, belief in the existence of better alternatives, time and effort required, and concerns about digestate and cooking on organic waste. The results of the analysis of weaknesses share with the environment in Bangladesh in terms of the initial cost of establishing biogas units (Saha et al., 2022) and in terms of the effectiveness of biogas technology to treat organic wastes (Iqbal et al., 2014).

Table 6 shows an analysis of the opportunities dimension in the SWOT variable:

**Table 6:** Formulation of opportunities points for the adoption of biogas technology in Syria

	Factor code	Description	Mean score (5-point Likert scale)*
Opportunities	O1	Environmental impacts of biogas technology	4.20
	O2	The use of biogas is feasible economically and environmentally	4.06
	O3	Biogas technology is a suitable alternative to the energy source currently used	3.98
	O4	The financial benefit of technology to the family	3.96
	O5	The desire to collectively participate in the Biogas Management Committee	3.91
	O6	The desire for the biogas technology revenues to be distributed to the technology participants according to the participation rates	3.74
	O7	The desire to manage biogas technology through the government or its representative locally	3.70
	O8	The desire to manage the use of biogas technology through a joint stock company	3.51
	O9	The desire for the revenue from biogas technology to be distributed equally to the villagers	3.17
	O10	Biogas technology is locally available	2.95
	O11	The desire to manage the use of biogas technology through a private company	2.53
The total average			3.61

*1—lowest, 5—highest

The average response of the respondents to the opportunities dimension was 3.61, which is greater than 3. In general, most of the paragraphs were higher than the neutral point of the Likert scale. These results identify the most critical opportunities in applying biogas technology in the areas studied.

Given the content of the description of the factors, the most important opportunity that should be taken care of is the awareness of the studied environment of the positive effects of technology on the environment, their knowledge of its economic feasibility as an essential and alternative source of traditional energy and its significant material effects, and the incubator's desire to participate in the management of the technology. The importance of the agricultural sector as a backbone of the Syrian economy (Aw-Hassan et al., 2014), with the presence of thousands of farm engineers and extension units in every township of the countryside, explains posed opportunities.

Table 7 shows an analysis of the dimension of the threat in the SWOT variable:

**Table 7:** Threats points formulation for the adoption of biogas technology in Syria

	Factor code	Description	Mean score (5-point Likert scale)*
Threats	T1	Taxes	4.16
	T2	Call for the governmental subsidies for biogas unit construction	3.98
	T3	Fees	3.92
	T4	Fear of inability to maintain and repair a biogas unit	3.64
	T5	Fear of lacking expertise in biogas unit's operation and maintenance	3.58
	T6	Biogas technology can harm the environment in which I live	2.18
The total average			3.59

*1—lowest, 5—highest

As shown in Table 6, the respondents' average response to the threat dimension was 3.59. Generally, most of the paragraphs were higher than 3. These results identify the most critical threats facing applying biogas technology in the areas studied.

Given the content of the factors description, the most critical threats that must be addressed are tax deductions, fees for establishing biogas, maintenance and lack of experience in dealing with technical difficulties. Similar threat dimensions in Brazil regarding the adopting biogas in the southern part of Brazil are related to the specific regulation regarding renewable energy support (Sacco et al., 2022). The SWOT matrix (Table 8) comprises only the first five scored statements in of internal (strengths and weaknesses) and external (opportunities and threats) factors.

**Table 8:**
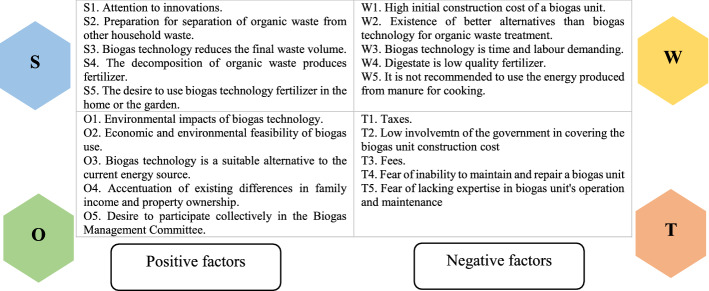
The SWOT Matrix

Our findings are in line with the study by Gottfried et al. (2018) on the material benefit of technology for household families and the desire for collective participation in the biogas management committee, and the strengths in terms of interest in innovations, as well as weaknesses over a long time, to invest in this technology, and in terms of opportunities through financial benefit and threats through high construction costs.

However, a study by Mukeshimana et al. (2021) showed that seven independent strategies have the most substantial ability to affect the entire renewable energy sector. Then, four strategies have the most significant driving force, such as increasing investment in renewable energy, providing incentives and policy support, creating favorable conditions for private investment and strengthening institutional management. This is consistent with our findings.

A study by Obrecht et al. (2011) proves that biogas technology reduces the final waste volume, and the decomposition products of the organic waste constitute fertilizer for plants and the desire to use the fertilizer produced by biogas technology.

Schaper et al. (2010) in their study demonstrated how a SWOT analysis of the most important factors shaped recommendations for farmers and extension services. This study aligns with ours in many ways, including the willingness for organic waste separation from the rest of home waste. Biogas technology reduces the final volume of trash and opportunities. These include the positive environmental impacts of biogas technology, the perception of biogas technology as an appropriate alternative to the currently used energy source, the financial benefit of the technology to the family, and the desire for collective participation in the biogas management committee.

Martin (2015) used the SWOT analysis to understand the gap between potential and the perspectives of biogas producers to understand the factors influencing biogas expansion in Sweden. The factors involved the availability and competition (consistent with our study in terms of threats), handling of digesters (consistent with our study in terms of threats), regulations, market incentives and support biogas production (consistent with our study in terms of opportunities).

### Analyzing alternatives using analytic hierarchy process

The AHP was used to scale experts' assessment of SWOT analysis results to determine the most important criteria to be focused on in the process of biogas technology adoption and the central region that gained the highest importance among other alternative criteria to specify the best areas to invest in biogas technology (Table 9). As a result, three areas were chosen to establish a biogas unit; we define these as follows:

1. Southern Region: Damascus (105 km^2^), Damascus countryside (18,032 km^2^), Daraa (3730 km^2^), and As-Suwayda (5550 km^2^); the sample of 102 surveyed farms (40% of the total sample).
2. Central Region: Hama (8,883 km^2^) and Homs (42,223 km^2^); the sample of 69 surveyed farms (27% of the total sample).
3. Coastal region: Lattakia (2297 km^2^) and Tartous (1892 km^2^); the sample of 84 surveyed farms (33% of the total sample).

**Table 9:** The average of the corresponding criteria for studied regions

The standard	Southern region	Central region	Coastal region
	Response weight	Response weight	Response weight
The level of the respondent's knowledge on biogas technology (M1)	4.04	4.00	3.87
The degree of biogas technology acceptance and potential use (M2)	3.98	3.95	3.91
The respondent’s approach to the use of biogas and digestate (M3)	4.13	4.06	3.98
Administrative aspects (M4)	4.12	4.02	3.85
Financial aspects (M5)	4.11	4.01	3.83
The average response rate of each region %	81.53	80	77.76

*1—lowest, 5—highest

**Fig. 1:**
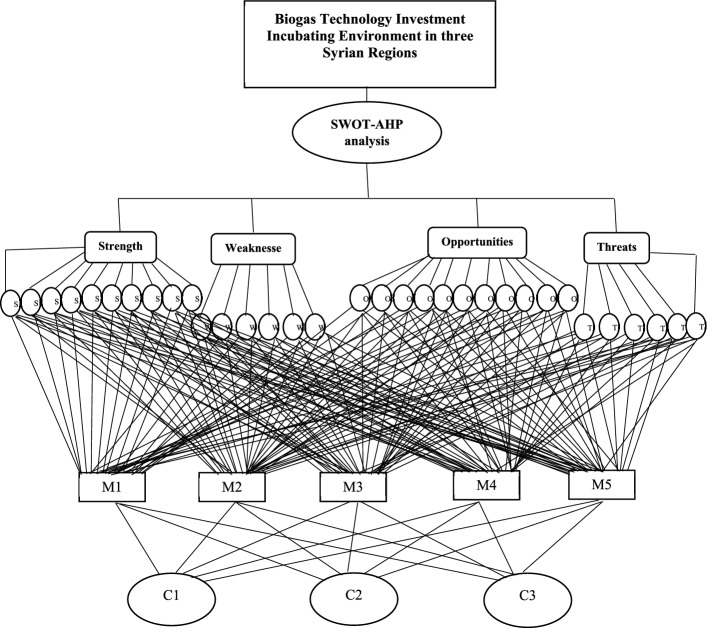
Hierarchical SWOT-AHP model

The criteria with the highest weight among each region are the approach to the use of biogas and digestate. The average response rate of the Southern region was 81.5%, while the average response rate of the Central Region was 80%. The average response rate of the Coastal region was 77.8% (Fig. 1).

Table 10 shows expert criteria, of which the highest ranked was the standard M3 (the approach for the use of biogas and digestate) at 46% of importance, followed by standard M4 (Administrative aspects) at 23%, Standard M5 (Financial aspects) at 20% and then the criterion M1 (the respondent’s knowledge about biogas) at 7% and finally M4 (the respondent’s acceptance and potential use of biogas) at 4.3%. The consistency ratio CR is 7% which is acceptable (not more than 10%). Whereas, for example in Rwanda, the hierarchy of criteria in terms of importance is as follows: financial, institutional, technical and socio-cultural barriers (Mukeshimana et al. (2021). In rural India (Yadav et al., 2022), the AHP analysis revealed the highest importance of economic dimension, then market, high installation cost, high competition from available fuel for free, capital subsidy, and the lack of easy loans (Table 11).

**Table 10:** Matrix of binary comparisons of the main criteria that affect the adoption of biogas technology

	M1	M2	M3	M4	M5	Weight
M1	1	2	0.14	0.2	0.5	0.07
M2	0.5	1	0.11	0.14	0.33	0.04
M3	7	9	1	2	3	0.46
M4	5	7	0.5	1	0.5	0.23
M5	2	3	0.33	2	1	0.20
CR = 0.07

**Table 11:** Matrix of binary comparisons of alternative regions to establish biogas units

	Southern	Central	Coastal	Weight
Southern	1	2	3	0.55
Central	0.5	1	1.5	0.27
Coastal	0.33	0.67	1	0.18
CR = 0.0				

**Fig. 2:**
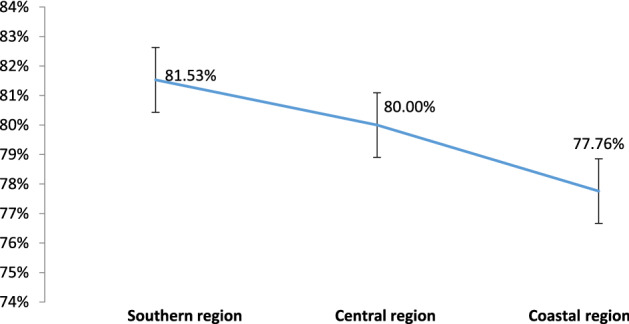
Alternative weights of the regions studied

The expert choice program was applied to demonstrate the alternatives (Fig. 2).

Results of binary comparisons matrix show that the most suitable region for investments in biogas technology is the southern region with 54.5%, followed by central (27.3%) and coastal (18.2%). The CR that equals to 0 shows complete stability in decision-making. Given that the primary feedstock is animal manure, the result of the investigation is consistent with the reality in terms of the concentration of livestock numbers and the amount of organic waste in Syria (CBS, 2019).

**Fig. 3:**
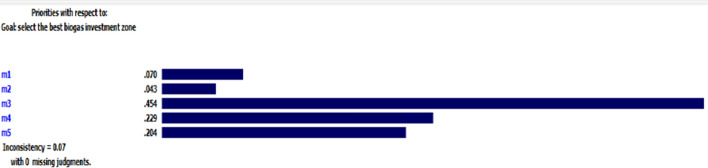
The marital comparisons of the main criteria that affect biogas technology adoption

The expert choice program was used to select the best region to invest in biogas technology (Fig. 3).

**Fig. 4:**
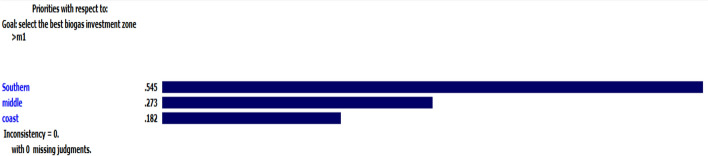
The marital comparisons of alternative regions to establish biogas units

The use of AHP in determining and evaluating the geographical suitability of biogas production at industrial level was used by Zhang et al. (2022) in China by dividing the 31 areas under study into three principle categories based on the following four criteria: societal and economic conditions, resources and environmental pressures. Results showed that the level of development achieved the highest importance among other alternative criteria. As similarly approached by Falcone and Sica (2019) in Italy, where the authors concluded that it is also essential to involve the implementation of a green agenda at both national and international levels when considering successful societal transitions in the field of green energy sector. This is even more pressing issue in the post-COVID-19 era (Giganti and Falcone, 2022; Roubík et al., 2022). The study by Akther et al. (2018) used environmental, social, safety and economic factors to analyze the criteria influencing the selection of a suitable location for the establishment of large-scale biogas units for the treatment of municipal waste in Bangladesh. However, AHP employed by De Jesus et al. (2021) to identify the appropriate areas to establish biogas units in southern Brazil used only geographical criteria (nearness to roads, proximity to pipes, proximity to organic waste suppliers) (Fig. 4).

## Conclusions and recommendations

SWOT-AHP analysis is conducive to providing the dimensions and factors that affect the investment in biogas technology and location selection in the Coastal, Central, and Southern regions of Syria. Exploiting opportunities based on available strengths will be the optimal strategy. The acceptance of biogas technology by Syrian society and the intention to use it will create awareness of its material and moral benefits, which will eventually lead to an increase in private investments in biogas plants. Furthermore, the interest of the community in innovations is one of the most critical strengths of adopting biogas technology. However, the positive impacts on environment and microeconomy are the main opportunities. On the contrary, the most outstanding weaknesses that hinder the application of biogas technology are the high costs, while the most critical threats are taxes and fees that can affect farmers' decision to establish biogas plants. Therefore, calls for governmental support on tax exemption and loan facilitation for farmers to adopt renewable energy projects are crucial in post-conflict times. The SWOT analysis results have been categorized into five main criteria; the approach to use biogas for energy and digestate as fertilizer was the best among the criteria in the study of the location of a biogas unit, followed by the respondent's acceptance and intended use of biogas technology, which was essential in making a decision toward investment in biogas technology.

According to the weight of alternative criteria for each region, the region with the highest percentage of alternative criteria is the southern region.

The study highlights the need to provide a clear strategy from the relevant authorities in the field of biomass-based energy and the need for awareness programmes to support the spread of biogas technology in rural areas as an ideal solution to produce energy from organic waste.

The main limitation of this study is that it does not take into account the northern and eastern parts of Syria due to the unstable situation there at the time of the search.

We suggest expanding the search for the best sites for the establishment of biogas units using geographic information systems (GIS) as an effective research methodology. The study focused on determining the criteria that affect biogas investment and the best areas to invest in this type of renewable energy. The expansion of research related to other types of sustainable energy can play an important role in improving the energy situation, especially in the post-war period.

## Declarations

### Data availability statement

The datasets generated during and/or analyzed during the current study are available from the corresponding author on reasonable request.
